# Virologic suppression and mortality of patients who migrate for HIV care in the province of British Columbia, Canada, from 2003 to 2012: a retrospective cohort study

**DOI:** 10.1186/s12913-015-1042-6

**Published:** 2015-09-14

**Authors:** Viviane Dias Lima, Nicola Goldberg, Lillian Lourenço, William Chau, Robert S. Hogg, Silvia Guillemi, Rolando Barrios, Julio S G Montaner

**Affiliations:** British Columbia Centre for Excellence in HIV/AIDS, 608-1081 Burrard Street, Vancouver, BC Canada V6Z 1Y6; Division of AIDS, Department of Medicine, Faculty of Medicine, University of British Columbia, Vancouver, BC Canada V6Z 1Y6; Faculty of Medicine, University of Toronto, Toronto, ON Canada M5S 1A8; Faculty of Health Sciences, Simon Fraser University, Burnaby, BC Canada V5A 1S6

## Abstract

**Background:**

Migration among persons living with HIV (PLWH) seeking HIV care is common; however its effect on health outcomes in resource-rich settings is not well understood. We conducted a retrospective cohort study to quantify the extent to which PLWH are migrating for care within British Columbia (BC) and its association with virologic suppression and mortality.

**Methods:**

Eligible PLWH first initiated treatment in BC between 2003 and 2012 (*N* = 3653). Analyses were performed at the regional Health Authority (HA) level (*N* = 5). For privacy reasons, we kept the name of these HAs anonymous and we re-named these five regions as 1 to 5. PLWH were classified according to the HA where they resided and received HIV care. We calculated all-cause mortality rates, life expectancies (at age of 20 years), and in, out and net migration rates across HAs using different demographic methods. Virologic suppression (<50 copies/mL) was based on the last viral load available for each PLWH. We also calculated per-capita rates (per 100 PLWH ever on cART) for each HA by dividing the number of PLWH by the number of physicians attending this population.

**Results:**

There is considerable heterogeneity in physician availability across all HAs, with per-capita rates (per 100 PLWH ever on cART) ranging from 2.2 (HA 1) to 12.7 (HA 3) based on the HA PLWH received care. We observed that in HAs 1, 4, and 5, between 4 and 10 % of PLWH migrated to HA 3 (i.e. the largest urban center) to receive care, and for HA 2 this proportion increased to 21 %. In HA 3, 77 % of its PLWH residents remained in the same HA for their care. Migrating to a larger center for HIV care was not associated with higher rates of viral load suppression; it was significantly associated with lower mortality rates and higher life expectancies.

**Conclusions:**

A thorough understanding of the reason(s) for these significant migration rates across BC will be critical to inform resource allocation and optimize the impact of HIV treatment.

## Background

In the early days of combination antiretroviral therapy (cART), HIV treatment options and access was limited and treatment guidelines were relatively complex [1]. As a result, HIV care was resource intense and required a high level of provider expertise; as such, services tended to be concentrated in specialized clinics in large urban centres. Since then, cART has evolved substantially, with an increased number of simplified, safer, better-tolerated and more effective treatment options [2]. The evolution of cART has made the goal of lifelong virologic suppression a realistic possibility, and has enabled the emergence of a bold new target for the global control of HIV/AIDS: the 90-90-90 Target [3]. In order to meet the 90-90-90 Target, persons living with HIV (PLWH) will necessitate regular access to an experienced care provider to fully adhere and benefit from treatment [4].

Since HIV is now considered a chronic manageable disease, therapeutic guidelines have been simplified and HIV care has increasingly shifted to decentralized community-based medical practices [5, 6]. Studies looking at patient outcomes for other chronic diseases have shown that access to community-based care offered by a continuous provider was associated with increased attendance at follow-up appointments and better disease control, which translated into fewer emergency department visits and hospitalizations, as well as decreased mortality [7–11]. Patients also reported higher satisfaction with their care when it is available locally and offered by a continuous provider or a team of providers [7, 11, 12].

The success that cART has achieved in improving disease outcomes among PLWH can be potentially hindered by patient migration, since migration has been identified as a possible source of loss to follow up in several studies, particularly in resource-limited settings [13–22]. Typically, it has been reported that PLWH tend to migrate towards urban centres following an HIV diagnosis to seek treatment and care [23]. It has been suggested that this migratory pattern is driven by the increased HIV stigma and the lack of access to specialized medical care in small communities [24, 25]. Regardless of the reason, migration has been associated with incomplete adherence to cART and higher mortality [26, 27]. However, the association of patient migration, especially when they routinely migrate for care, with cART outcomes in resource-rich settings is not well understood.

Migrating for care is particularly concerning given the existing evidence on the importance of community-based continuous care for improving other chronic disease outcomes. We, therefore, conducted the present retrospective cohort study to quantify the extent to which PLWH are migrating for their care within British Columbia (BC), Canada, and its association with HIV virologic suppression and all-cause mortality. We hypothesized that receiving community-based HIV care is associated with better health outcomes.

## Methods

### Data sources

Data from eligible PLWH were extracted from the BC Centre for Excellence in HIV/AIDS (BC-CfE) Drug Treatment Program monitoring and evaluation system. Since October 1992, the distribution of antiretrovirals in BC has been the responsibility of the BC-CfE. Antiretroviral drugs are distributed to all PLWH in BC according to specific guidelines generated by the Therapeutic Guidelines Committee. These guidelines have remained consistent with those put forward by the International AIDS Society-USA since 1996 [1, 2].

Eligible PLWH were cART naïve, ≥20 years old, enrolled between January 1, 2003 and December 31, 2012 and followed: (1) until December 31, 2013 (if alive); (2) until the last contact date if they were lost to follow-up or if they moved out of BC); or (3) until the date of death. These individuals started cART consisting of two nucleoside reverse-transcriptase inhibitors as backbone, plus either a non-nucleoside reverse-transcriptase inhibitor or a boosted protease inhibitor. They must also have had a CD4 count and plasma viral load measurement within six months of the initial antiretroviral date.

BC is a geographically large province with major regional differences in population demographic and socio-economic characteristics, and in health needs. As such, the government divided the province in five health authorities (HA) [28], which are responsible for the management and delivery of health services in geographically defined subpopulations in BC. Typically, in the BC-CfE, physicians monitor PLWH on cART at intervals no longer than three months, at which time, prescriptions are renewed or modified, and PLWH’ addresses are updated in the BC-CfE database. Every year, physicians are asked to update their addresses at the College of Physicians and Surgeons of BC, and their addresses are continuously updated in our database. In this study, PLWH and their physician addresses were then used to classify individuals according to the five HAs where PLWH resided or where they received medical care.

To provide a context to the readers, the five BC HAs are heterogeneous regarding several socio-economic aspects including income, housing, employment, poverty, and ethnicity. These HAs are also heterogeneous in the way they deliver care to the BC population. In this study, we kept the name of these HAs anonymous for privacy reasons and we re-named these five regions as 1 to 5. Based on the latest Regional Socio-Economic Index calculated by the BC government, HA 3 had by far the highest and HA 5 the lowest socio-economic index, while the other three HAs had very similar intermediate socio-economic indexes. It is also noteworthy that HA 3 has the highest number of clinical staff, prescribing physicians, comprehensive HIV supportive services and it hosts the BC-CfE.

### Outcome variables

Crude, cART era and HA-specific all-cause mortality rates (per 1000 person-years of follow-up) were obtained for PLWH who have ever started treatment. These mortality rates were calculated dividing the number of deaths (all causes) by the number of person-years of follow-up; corresponding 95 % confidence intervals and test for the equality for these rates were based on the Fisher’s exact test [29]. Deaths occurring amongst PLWH during the follow-up period were identified on a daily basis from physician reports and through monthly record linkages carried out with the BC Vital Statistics Agency. Person-years for PLWH on treatment were calculated from the date of first cART to December 31, 2013, or the date of death or censoring.

cART era was defined according to the year of first treatment (2003–2007 and 2008–2012) and based on different treatment rollout periods in BC since 2003; with the most significant period being between 2009 and 2012 when the BC government funded the STOP HIV/AIDS initiative to further address the HIV epidemic in the province [http://stophivaids.ca]. HAs 3 and 5 were the regions targeted by this initiative during the study period. Age-specific mortality rates were used to calculate life expectancies, and they were specifically obtained for the age groups 20–29, 30–34, 35–39, 40–44, 45–49 and 50+ years to account for the age distribution of PLWH in our cohort. The construction of these life tables and their respective 95 % confidence intervals (CIs) were obtained as outlined in Chiang [30]. Additionally, we assessed whether these individuals were suppressed (i.e. a plasma viral load <50 copies/mL) based on the last viral load available during their follow-up. Note that all plasma viral load measurements in BC are centrally done at the St Paul’s Hospital virology laboratory.

To correct the data for loss to follow-up, we built an explanatory multivariable logistic regression model (modeling the probability of death) among individuals whose status at the end of follow-up was either alive or dead. The final model contained the variables adherence, age, year of first cART, follow-up time, baseline CD4 cell count and plasma viral load. The area under the receiver operating characteristic curve (AUC-ROC), a measure of goodness of fit measuring the model’s ability to discriminate between those PLWH who died and those who did not, ranges from 0.50 (no discrimination) to 1.00 (perfect discrimination) [31]. In this model, we obtained an AUC-ROC of 0.91, which is considered an outstanding discrimination. Therefore, we applied the estimated model coefficients to the data of PLWH who were lost to follow-up to calculate their respective probability of being dead by the end of follow-up. The cut-off used for these estimated probabilities was one and, in this case, only PLWH whose estimated probability was equal to one were considered dead at the end of follow-up. Given that these individuals do not have an exact date of death, we considered their last contact date as the date of death.

### Migration indicators

For the migration analyses, we first calculated per-capita rates (per 100 PLWH ever on cART) for each HA by dividing the number of PLWH by the number of physicians attending this population. This measure describes the heterogeneity in physician availability across the different HAs. We then defined migration for care as the movement from the HA where the PLWH resided to the HA that he/she received care. Note that this movement (within short and long distances) is temporary and may be for voluntary or involuntary reasons. A number of migration indicators were used to study the trends across HAs during 2003–2012 [32, 33]. First, we assessed internal migration of different HAs by comparing the HA where they received care to the HA where the PLWH resided. Second, we calculated three common migration rates for each HA defined as:

- In-migration rate: $\frac{I_{HA\left[i\right]}}{P_{HA\left[i\right]}}\times 1000$, where *I*_*HA*[*i*]_ is the number of in-migrants during a specified time for the *i*^*th*^ HA (*i* = 1, …,5) and; *P*_*HA*[*i*]_ is the population of the *i*^*th*^ HA at the mid-point of the migration interval. In lay terms, in this study, the in-migrants are the ones who leave the HA that they resided to receive care in another HA.
- Out-migration rate: $\frac{O_{HA\left[i\right]}}{P_{HA\left[i\right]}}\times 1000$, where *O*_*HA*[*i*]_ is the number of out-migrants during a specified time for the *i*^*th*^ HA (*i* = 1, …,5) and; *P*_*HA*[*i*]_ is defined above. In lay terms, in this study, the out-migrants are the ones arriving from different HAs to receive care in the HA that is not their residence.
- Net-migration rate: $\frac{I_{HA\left[i\right]}-{O}_{HA\left[i\right]}}{P_{HA\left[i\right]}}\times 1000$, where *I*_*HA*[*i*]_, *O*_*HA*[*i*]_ and *P*_*HA*[*i*]_ are defined above.

All analyses were performed using SAS software version 9.3 (SAS, Cary, NC). The BC-CFE received approval for this study from the University of British Columbia ethics review committee at the St Paul’s Hospital, Providence Health Care site (P05–123). The study complies with the BC’s Freedom of Information and Protection of Privacy Act. The study was conducted primarily using anonymized administrative databases, and therefore informed consent was not required.

## Results

### Baseline characteristics

This study was based on data of 3653 PLWH who initiated cART for the first time in BC between 2003 and 2012. The baseline characteristics, summarized in Table 1, show that the majority of PLWH were male (82 %), had age between 25 and 44 years, initiated cART between 2009 and 2012, had baseline CD4 cell count between 50 and 349 cells/mm^3^ and plasma viral load greater or equal to 5.00 log_10_ copies/mL. The median follow-up of these individuals was 4.5 years (25^th^ – 75^th^ percentile: 2.4 – 6.8 years).

**Table 1:** Characteristics of the study population at the start of combination antiretroviral therapy from 2003 to 2012 in British Columbia

Covariates and outcomes	Frequency (%) or
	Median (Q_1_ - Q_3_)
Gender	
Male	2979 (82 %)
Female	674 (18 %)
cART era	
2003 - 2007	1554 (43 %)
2008 - 2012	2099 (57 %)
Age (years)	
20 - 24	109 (3 %)
25 - 44	2138 (59 %)
45 - 64	1324 (36 %)
65 +	82 (2 %)
CD4 cell count (cells/mm^3^)	
<50	421 (12 %)
50 - 199	1154 (32 %)
200 - 349	1172 (32 %)
350+	906 (25 %)
Viral load (log_10_copies/mL)	
<5.00	1545 (42 %)
5.00+	2108 (58 %)
Adherence to therapy during the first year on cART (%)	
≥95 %	2464 (67 %)
80 - <95 %	501 (14 %)
40 - <80 %	468 (13 %)
0 - <40 %	220 (6 %)
Follow-up (person-years)	4.50 (2.40 - 6.80)

*cART* combination antiretroviral therapy; Q_1_ stands for 25^th^ percentile; Q_3_ stands for 75^th^ percentile

### Migration patterns

**Fig. 1:**
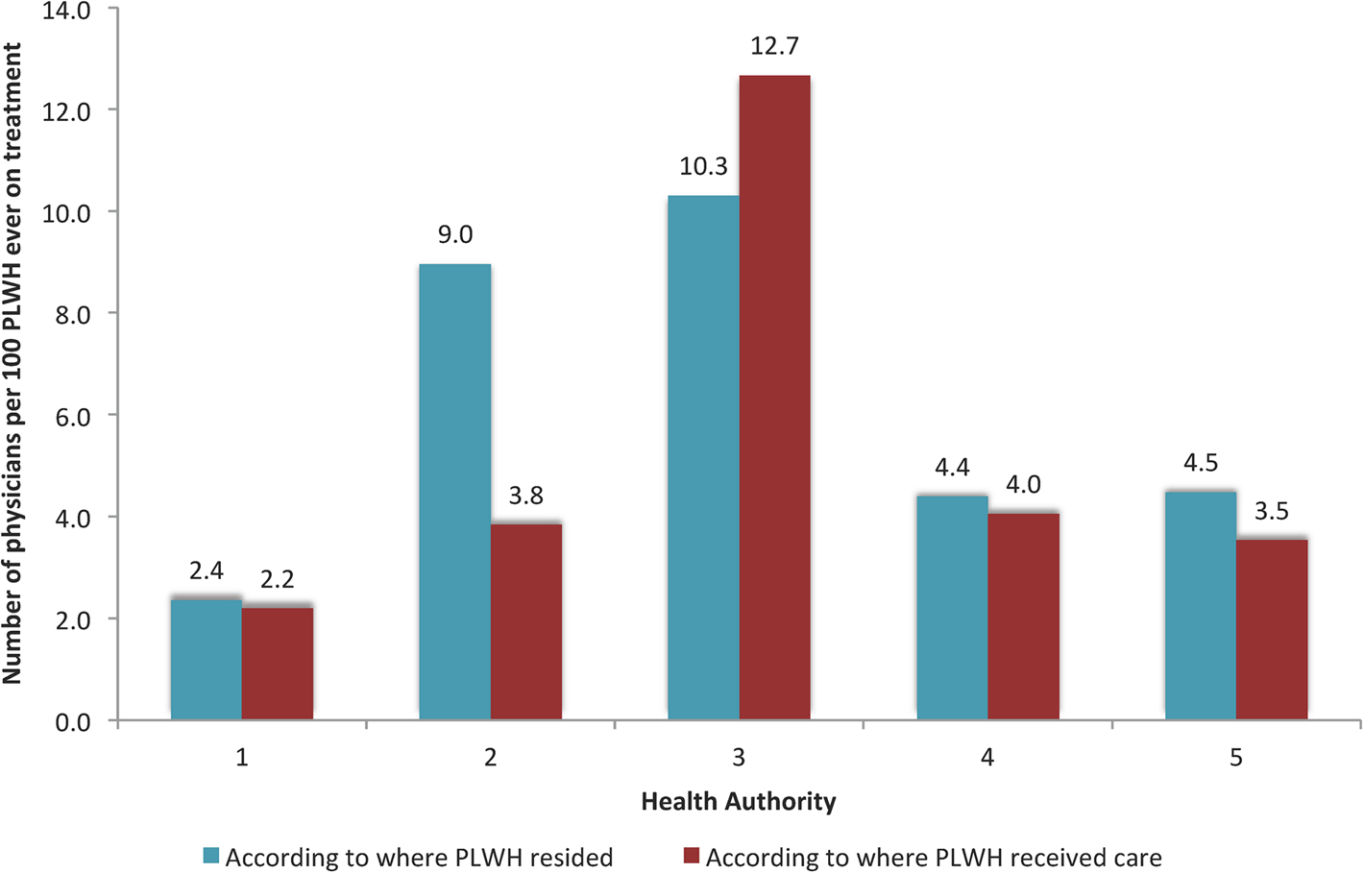
Physician per capita rates by Health Authority in British Columbia from 2003 to 2012. PLWH stands for persons living with HIV

There is considerable heterogeneity in physician availability (i.e. per-capita rates per 100 PLWH ever on cART) across all HAs. As illustrated in Fig. 1, when physician availability was calculated according to the HA of PLWH residence, for every one physician, HA 1 had approximately 2.4 PLWH, HA 2 had 9.0, HA 3 had 10.3, HA 4 had 4.4 and HA 5 had 4.5. In contrast, when physician availability was calculated using the HA where PLWH received care, the per capita rates slightly dropped in all HAs with the exception of HA 2 which significantly dropped to 3.8 (57 % decrease), HA 5 which dropped to 3.5 (21 % decrease) and HA 3 which increased to 12.7 (23 % increase).

**Fig. 2:**
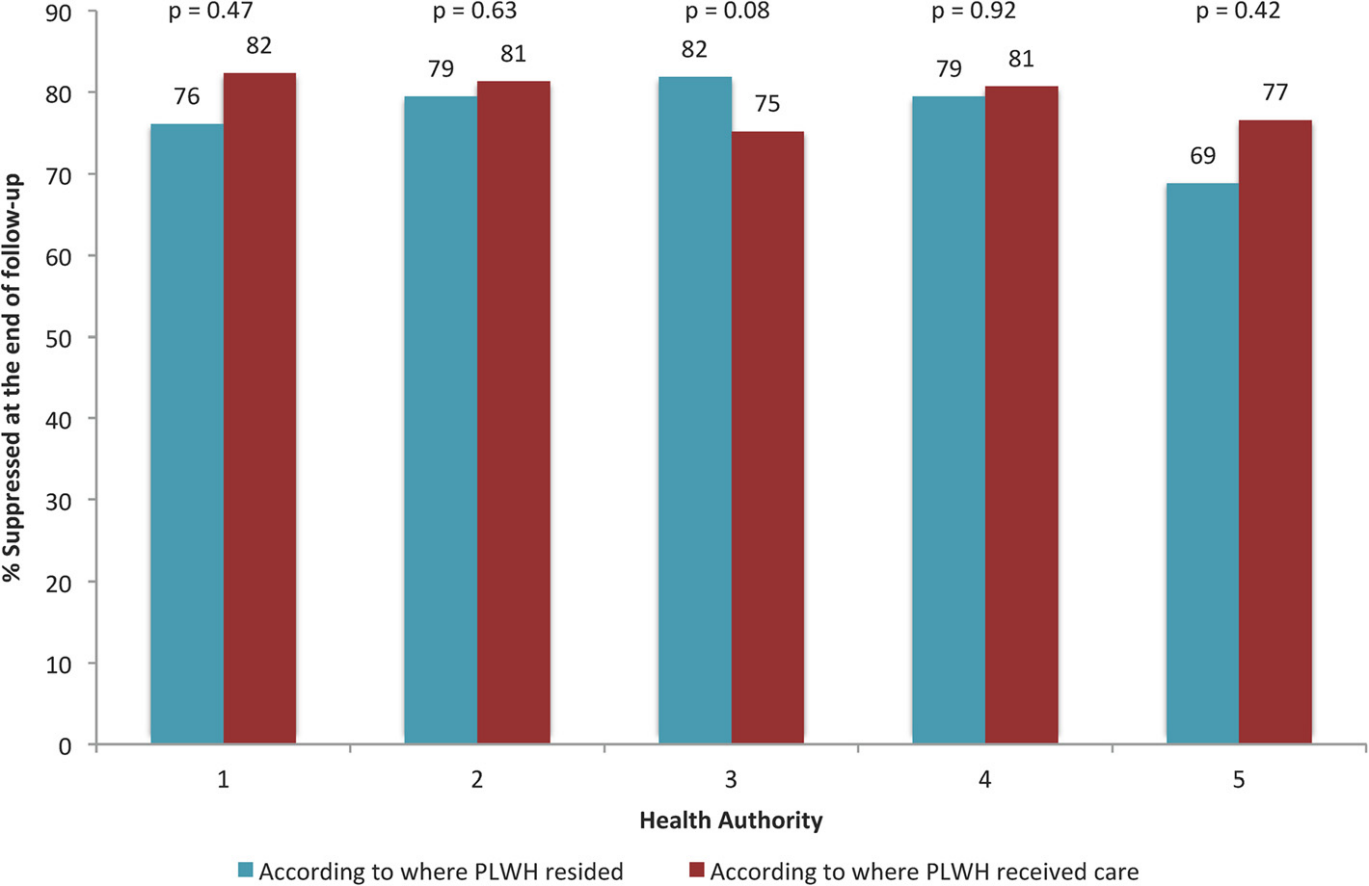
Distribution of PLWH according to suppression status at the end of follow-up by Health Authority in British Columbia from 2003 to 2012. PLWH stands for persons living with HIV

Table 2A shows the distribution of PLWH according to their residence and to where they received care during the study period. We observed that in HAs 1, 4, and 5, between 4 and 10 % of PLWH migrated to HA 3 (i.e. the largest urban center) to receive care, and for HA 2 this proportion increased to 21 %. In HA 3, 77 % of its PLWH residents remained in the same HA for their care. Consequently, we obtained a negative net-migration rate of −70.0, −75.8, and −248.2 per 1000 PLWH on cART for the HAs 1, 4 and 5, respectively, indicating that PLWH were leaving these HAs at a higher rate than staying in them (Table 2B). HA 3 had a net-migration rate of 186.6 per 1000 PLWH on cART showing that this HA is absorbing most PLWH migrating for care across BC. Interestingly, HA 2 had the highest negative net-migration rate (−1.334.4), with most of its PLWH residents leaving to receive care in HA 3.

**Table 2:** Inter-Health Authority migration rates per 1000 population in British Columbia from 2003 to 2012

Health Authority where PLWH received care	Health Authority where PLWH resided					
	1	2	3	4	5	Total
1	145	6	39	6	1	197
2	11	231	477	10	4	733
3	19	65	2,027	37	6	2,154
4	3	5	75	299	1	383
5	6	7	30	4	129	176
Total	184	314	2,648	356	141	3,643
Health Authority	Population at risk	In-Migrants	Out-Migrants	In-migration rate	Out-migration rate	Net-migration rate
1	184	39	52	212.0	282.6	−70.7
2	314	83	502	264.3	1,598.7	−1334.4
3	2,648	621	127	234.5	48.0	186.6
4	356	57	84	160.1	236.0	−75.8
5	141	12	47	85.1	333.3	−248.2

*PLWH* stands for persons living with HIV

### Migration and HIV outcomes

At the end of follow-up, we observed 421 deaths during 17430 person-years for a crude mortality rate 24.15 per 1000 person-years (95 % confidence interval (CI) 21.90 – 26.57). A total of 666 (18 %) PLWH were lost to follow-up, and of these, 157 (24 %) were LTFU because they moved out of BC. After correcting the data for loss to follow-up, we re-coded 77 PLWH as being dead at the end of follow-up. Thus, during follow-up there were 498 deaths for a (adjusted) crude mortality rate 28.57 per 1000 person-years (95 % CI 26.12 – 31.19), and corresponding life expectancy (at the age 20 years) of 34.53 years (standard error 1.09) (Table 3).

**Table 3:** Mortality rates and life expectancies for PLWH who have ever initiated combination antiretroviral therapy from 2003 to 2012, stratified by Health Authority

Mortality outcomes	Population size	Observed deaths	Follow-up (person-years)	Mortality rate (95 % CI) per 1000 person-years	Life expectancy at age 20 years (standard error)
British Columbia	3653	498	17430	28.57 (26.12 − 31.19)	34.53 (1.09)
Health Authority where PLWH resided					
1	198	36	937	38.42 (26.91 − 53.19)	32.96 (2.82)
2	735	94	3591	26.18 (21.15 − 32.03)	34.36 (2.67)
3	2155	266	10294	25.84 (22.83 − 29.14)	37.66 (1.53)
4	386	66	1931	34.18 (26.43 − 43.48)	33.50 (2.54)
5	179	36	678	53.08 (37.19 − 73.51)	19.41 (3.38)
Health Authority where PLWH received care					
1	184	32	885	36.15 (24.73 − 51.05)	34.74 (2.96)
2	314	45	1447	31.09 (22.68 − 41.61)	34.11 (3.87)
3	2648	316	12781	24.72 (22.07 − 27.61)	37.32 (1.35)
4	356	68	1739	39.10 (30.37 − 49.57)	33.40 (2.20)
5	141	33	524	62.99 (43.35 − 88.44)	17.35 (3.67)

*PLWH* stands for persons living with HIV

As illustrated in Fig. 2, there was no statistically significant difference in suppression status among PLWH according to whether or not they received care in the HA of residence. We also calculated the mortality rate (per 1000 person-years) and life expectancy (at age 20 years) for each HA according to the patient and physician address during 2003–2012 (Table 3). When we used the patient’s residence, HA 5 had the highest mortality rate (53.08) and the lowest life expectancy at age 20 years (19.41); HA 3 has the lowest mortality rate (25.84) and the highest life expectancy at age 20 years (37.66) in the province. When we classified PLWH according to the HA they received care, HA 5 was the only region that experienced the highest increase in mortality rate (62.99; 19 % increase) and a higher decline in life expectancy at age 20 years (17.35; 11 % decrease). We found that HAs 1, 2, 3 and 4 had similar life expectancies regardless of where PLWH lived or sought care; however the same was not seen when we looked at the mortality rates in each of these regions. While the mortality rate in HAs 1 and 3 decreased to 36.15 (6 % decrease) and 24.72 (4 % decrease), respectively, the mortality rate in HAs 2 and 4 increased substantially to 31.09 (19 % decrease) and 39.10 (14 % decrease), respectively.

**Fig. 3:**
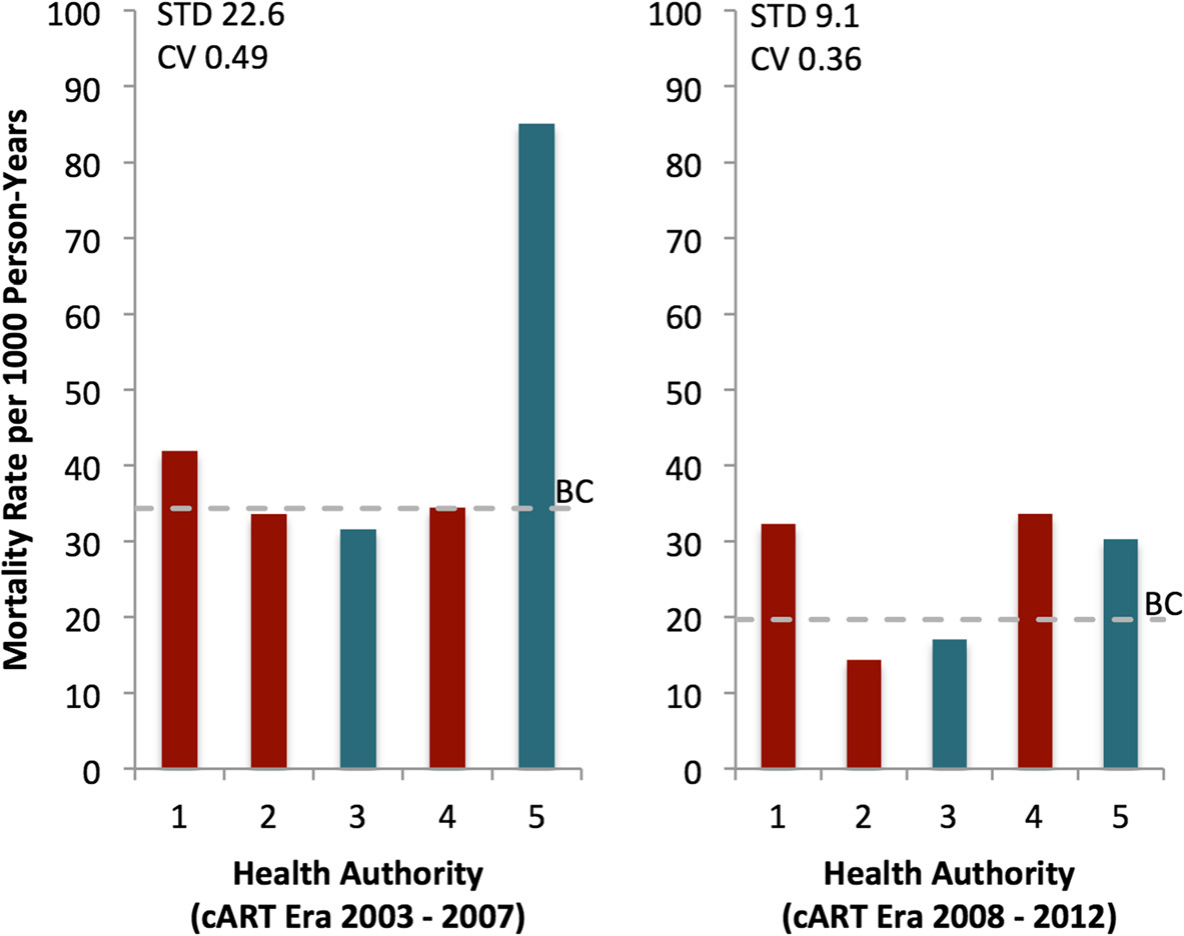
Mortality rate by combination antiretroviral therapyera and by the Health Authority where the patient received care in British Columbia from 2003 to 2012. cART combination antiretroviral therapy; stands for PLWH stands for persons living with HIV; STD stands for standard deviation (higher values means more variation); CV stands for coefficient of variation (higher values means more variation). Red bars represent the Health Authorities that were not targeted by the intervention STOP HIV/AIDS, and green bars represent the Health Authorities targeted by this intervention

Figure 3 presents trends in mortality rates according to the cART expansion eras from 2003 to 2012. This figure also highlights the regions that were targeted by the STOP HIV/AIDS initiative (in green) and shows that the heterogeneity in mortality rates across BC has decreased over time especially during 2009–2012 (coefficient of variation ranged from 0.49 (in 2003–2007) to 0.36 (in 2008–2012)). This decline was even more pronounced in the HAs initially targeted by the STOP HIV/AIDS initiative, with the mortality rate in HA 3 dropping from 31.58 (in 2003–2007) to 17.01 (in 2008–2012) per 1000 person-years (46 % decrease; *p*-value <0.0001); and HA 5 from 85.14 (in 2003–2007) to 30.28 (in 2008–2012) per 1000 person-years (64 % decrease; *p*-value = 0.0041). We also observed a significant decrease in HA 2 from 33.61 (in 2003–2007) to 14.40 (in 2008–2012) per 1000 person-years (57 % decrease; *p*-value = 0.0005).

## Discussion

Our results demonstrate that there is a substantial heterogeneity in the number of PLWH seeking medical care outside the HA where they resided; between 4 and 21 % of PLWH in each HA migrated to HA 3, which hosts the province’s largest HIV care center. Although we investigated whether out-migration rates in these regions were due to physician availability, we observed that physicians working outside HA 3 were caring for fewer PLWH as many of their patients were seeking care in HA 3. Additionally, we assessed whether or not this migration for care was associated with health outcomes. We observed that while migrating to a larger center for HIV care (i.e. HA 3) was not associated with higher rates of viral load suppression; it was significantly associated with lower mortality rates, and slightly higher life expectancies. Finally, during the study period, we demonstrated that mortality rates decreased significantly in HAs 2, 3 and 5.

We hypothesized that receiving community-based HIV care would be associated with better outcomes, given the previous evidence from other chronic diseases which suggested that access to a local, continuous care provider was associated with better adherence to treatment, follow-up, and overall health [7–11]. Our findings are, therefore, in direct contrast to our initial hypothesis. There are a number of possible explanations for these discrepancies. First, there are potential confounding variables that may explain the association between migration and decreased mortality, such as the differential in socio-determinants of health across HAs in BC. Additionally, it is common for PLWH to move to regions with larger centers for HIV care when they are really sick, and for these individuals to move back to their originating regions when they are close to dying [34]. Second, we assumed that the lower mortality associated with receiving care in the largest urban center in BC was mostly due to the larger availability of medical experts. It is important to mention that HA 3 also offers many non-medical services for PLWH, such as housing, addictions and psychological support, as well as, support groups and peer networks. These other medical and non-medical services may partially explain the better outcomes seen in HA 3. Further studies will be needed to fully understand the reasons for this type of migration in BC.

Our study has a number of important strengths. There are very few studies in resource-rich settings examining the association of migration among PLWH and health outcomes [10, 34–39]. This is the first study to demonstrate the relationship between migrating for HIV care and mortality during the modern cART era and within a treatment rollout program in BC. Also, we looked at a large sample of PLWH receiving care within a province-wide program, where all patients can access free medical attention, cART, and laboratory monitoring. Thus, it is unlikely that our results were influenced by sample size issues or biased by direct financial limitations to access medical care. It is also important to mention that delayed reporting of deaths was not likely a factor to influence our results, since most deaths were reported within one month through active follow-up with physicians and regular linkages to the BC Vital Statistics Agency. We also identified that a large number of PLWH are migrating to different HAs for care, and therefore, given that the life expectancy of PLWH is steadily increasing over time, the continual migration of these individuals can potentially overburden the resources of the receiving HAs. We believe that the results from this study can inform resource allocation in BC. Additionally, the methods used in this study can be further utilized to assess the impact of migration among PLWH in other settings around the world. Lastly, this study was possible since we maintain a state-of-art population-based database with detailed and up-to-date information on patient and physician addresses.

On the other hand, our study has a number of potential limitations. We did not control for physician experience since there is no gold-standard measure available for this purpose. We also did not expand our analyses to control for patient characteristics that may confound the relationship between migration for care and health outcomes. For example, it is well-known that for a patient to successfully supress their viral load, they must be diagnosed and initiated on cART in a timely basis, they need to maintain long-term optimal adherence to their treatment, and it is expected that they see their follow-up physician regularly to renew their cART prescriptions and monitor the safety and efficacy of their treatment [5]. The personal circumstances and attributes that allow a patient to meet these requirements, including financial resources, independence, and stability may have confounded our results. Additionally, these results may have been influenced by the perceived stigma/discrimination that some PLWH might experience in smaller communities [19, 25]. Another limitation in these analyses relates to 18 % of PLWH in our study being lost to follow-up. Although we adjusted our data by means of a highly predictive statistical model, we continue to conduct ongoing linkages with the BC Vital Statistics Agency to ascertain the health status of these individuals in our database. Finally, there are a number of ancillary non-medical services offered in larger treatment centres to PLWH that are not available in smaller centres, and these services are known to potentially impact health outcomes. However, it is unclear to what extent these services may explain the association between migration and study outcomes.

## Conclusions

The current study revealed that a significant number of PLWH are migrating for care towards larger urban centers in BC and that this migration was associated with decreased mortality. These findings are in contrast to the experience in other chronic disease areas, and as such have important implications for health policy and resource allocation. Further research will be required to identify what factors are associated with migration for care, and whether these factors are independently protective against mortality. A thorough understanding of these issues will be critical to inform resource allocation aimed at optimizing the impact of cART.
